# Liposomal Nanotraps Neutralize *Listeria monocytogenes* Toxins to Enhance Macrophage Viability and Antibacterial Capacity

**DOI:** 10.1097/IM9.0000000000000177

**Published:** 2025-04-17

**Authors:** Hervé Besançon, Margherita Polidori, Andrea Hostettler, Victor Nizet, Anna Oevermann, Eduard Babiychuk

**Affiliations:** 1 Institute of Anatomy, University of Bern, Bern, Switzerland; 2 Division of Host-Microbe Systems & Therapeutics, Department of Pediatrics, University of California, San Diego, La Jolla, California, USA; 3 Division of Neurological Sciences, Vetsuisse Faculty, University of Bern, Bern, Switzerland; 4 Graduate School for Cellular and Biomedical Sciences, University of Bern, Bern, Switzerland; 5 Skaggs School of Pharmacy and Pharmaceutical Sciences, University of California, San Diego, La Jolla, California, USA

**Keywords:** infection, toxin, liposome, nanotraps, *Listeria*, antivirulence, macrophage

## Abstract

*Listeria monocytogenes* is a human and veterinary pathogen, one of the most common agents of foodborne infections worldwide. It can cause severe complications such as meningitis or miscarriage. Anti-virulence therapies, which target virulence factors such as pore-forming toxins, offer an alternative approach to combating infections. In this study, cholesterol-containing liposomal nanotraps effectively neutralized *L. monocytogenes* exotoxins, particularly listeriolysin O (LLO), thereby protecting mammalian cells. Notably, toxin neutralization was observed under both neutral and acidic conditions, where LLO activity is optimized to facilitate bacterial escape from the phagosome. Liposomal nanotraps were phagocytosed by macrophages and colocalized with intracellular *Listeria*, increasing the clearance rate of intracellular bacteria. These findings expand the potential use of broad-spectrum liposomal nanotrap therapy, which could be employed alongside current standard of care treatments to assist the immune system in controlling virulent pathogens.

## Introduction

*Listeria monocytogenes* (Lm) is a virulent gram-positive foodborne pathogen causing significant mortality in humans and animals.^1^ Fatalities are predominantly due to meningitis and sepsis, particularly in neonates, immunocompromised individuals and pregnant women.^1,2^ Other symptoms include gastroenteritis, headache, and miscarriage or stillbirth.^1,2^ The rise of antibiotic resistance complicates treatment for many bacterial infections, and identification of resistant Lm strains proves that this pathogen is no exception.^3,4^ Farm animals, such as cattle, poultry and swine, which act as reservoirs for Lm, are frequently exposed to antibiotics, thereby promoting resistance.^5^

The increasing burden of Lm infections underscores the need for alternatives to traditional antibiotics. One promising approach is antivirulence therapy, which targets the neutralization of virulence factors that pathogens use to establish infections.^6,7^ A key advantage of antivirulence approaches is the reduced selective pressure for resistance, as they only target bacteria actively expressing virulence factors, leaving the broader bacterial population, including commensals, largely unaffected.^6,7^ Among the most critical virulence factors are pore-forming toxins (PFTs), which disrupt host cell membranes, leading to cell death by altering ion flow and cellular homeostasis. PFTs also facilitate tissue invasion and immune evasion by breaching epithelial barriers and killing immune cells.^8–10^ These toxins typically interact with specific lipids or proteins as binding receptors on host cell membranes.^11^

Liposomal nanotraps offer a novel means of neutralizing PFTs. The nanotraps are tailored artificially to contain high concentrations of receptors targeted by toxins.^12–14^ Without functional PFTs, pathogens are more easily cleared by the host immune system. Liposomal nanotraps have shown efficacy in capturing a variety of PFTs, providing protection in animal models and demonstrating safety in a human clinical trial.^12,14–16^

Lm produces the potent toxin listeriolysin O (LLO), a member of the cholesterol-dependent cytolysin (CDC) family.^17^ LLO is secreted as a 56-kDa soluble monomer that binds to host cell membranes, oligomerizes, and forms active pores.^18^ Extracellular LLO activity is essential for Lm internalization in nonphagocytic cells, such as epithelial cells, and for triggering apoptosis.^9,19,20^ Lm’s intracellular lifecycle relies on LLO to escape the phagosome and replicate in the host cell cytosol.^2^ LLO-deficient strains are avirulent due to their inability to escape before phagosome-lysosome fusion, which makes LLO a prime target for neutralization.^21–23^

Neutralizing LLO and other Lm toxins could protect host cells and immune defenses, helping limit bacterial replication and promoting effective adaptive immune responses.^24,25^ An optimal antitoxin therapy for Lm infections must neutralize toxins both extracellularly and within the phagosome, preventing bacterial escape into the cytosol. In this study, we present experimental evidence that cholesterol-containing liposomal nanotraps efficiently neutralize toxins from clinically relevant Lm strains, conferring protection to host cells. Notably, we demonstrate for the first time that this treatment is also effective against intracellular bacteria.

## Results

### Listeria monocytogenes hemolysins are neutralized by cholesterol-containing liposomes

**Figure 1:**
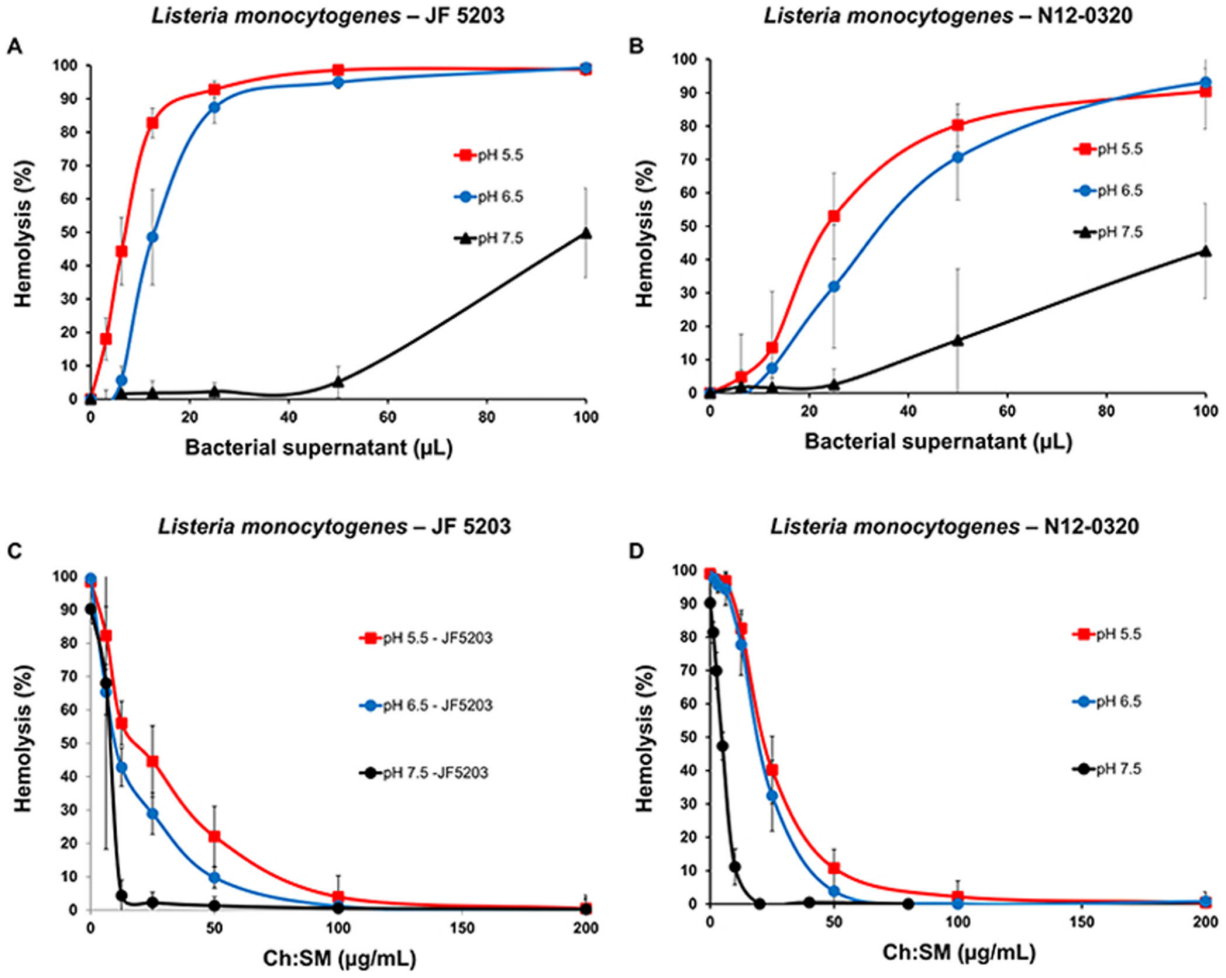
Lm hemolytic toxin activity was increased at acidic pH and neutralized by Ch:SM-liposomes. A and B: Acidic pH increased the hemolytic activity of Lm. C and D: The addition of Ch:SM-liposomes protected erythrocytes against Lm hemolysins at all pH values tested. The supernatant concentrations were adjusted to reach ~100% hemolysis at pH values of 6.5 and 5.5, while the highest assay-compatible concentrations were used at pH 7.5. Error bars = mean ± SD. N ≥ 3. Ch, cholesterol; Lm, *Listeria monocytogenes*; SM, sphingomyelin.

The human clinical Lm isolate (N12-0320, serotype 4b, meningitis) and the bovine veterinary Lm strain (JF5203, serotype ST1, brainstem encephalitis) were found to have significant hemolytic activity (Figure 1). LLO forms disulfide bonds, limiting its activity unless it is reduced.^26^ If LLO is primarily responsible for the observed hemolysis, we would expect little to no hemolysis in the absence of a reducing agent. This is indeed what we observed in the absence of the reducing agent dithiothreitol (DTT) (Figure S1A). The activity of LLO increases as the pH drops within the phagosome, facilitating phagosomal escape before phagosome-lysosome fusion.^26^ Therefore, we assessed hemolytic activity in the supernatant at pH values of 6.5 and 5.5, which simulate the early- and late-endosome environments, respectively.^27^ The hemolytic activity of the Lm supernatant significantly increased as the pH decreased from 7.5 to 6.5, but no further increase was observed at pH 5.5 (Figure 1A and B). In contrast, the activity of another CDC toxin, pneumolysin (PLY), was unaffected by pH changes (Figure S2). Our results reinforce that LLO, unlike other CDC toxins, is significantly adapted to function under acidic conditions.

Given the importance of LLO as a major Lm toxin and its strong affinity for cholesterol,^28^ we evaluated the protective potential of liposomes composed of cholesterol and sphingomyelin (Ch:SM). These Ch:SM-liposomes, which do not contain ionizable lipids typically found in pH-sensitive formulations, are expected to remain stable under acidic conditions. The results showed that Ch:SM-liposomes effectively neutralized Lm toxins at pH 7.5, 6.5 and 5.5 (Figure 1C and D), providing equivalent protection across these pH values. Although higher concentrations of liposomes are generally needed as pH decreases from 7.5 to 6.5 or 5.5, the variability in hemolytic activity made it difficult to quantify the exact difference. Notably, the protective effect was cholesterol-dependent, as liposomes composed solely of sphingomyelin (SM) were unable to reduce hemolysis (Figure S1B).

### Liposomal nanotraps protect a monocytic cell line challenged with Listeria monocytogenes toxins

**Figure 2:**
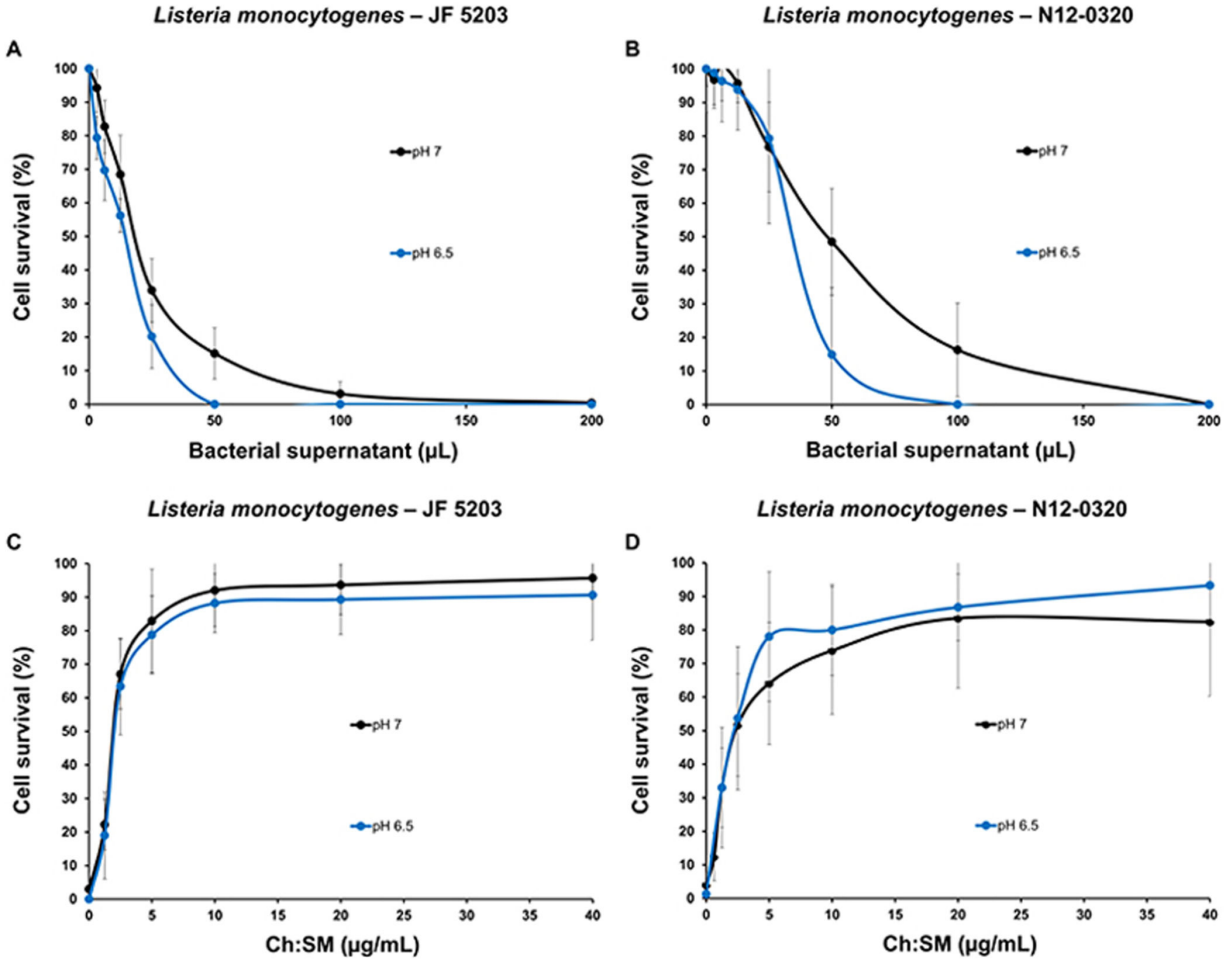
THP-1 cells were sensitive to Lm-secreted cytotoxins but the addition of Ch:SM-liposomes conferred protection. A and B: The Lm supernatant exhibited potent cytotoxicity toward monocytic THP-1 cells, with increased activity at pH 6.5 compared with 7.0. C and D: The addition of Ch:SM-liposomes protected THP-1 cells at both neutral and acidic pH. Supernatant concentrations were adjusted to reach ~100% cell death. Error bars = mean ± SD. N ≥ 3. Ch, cholesterol; Lm, *Listeria monocytogenes*; SM, sphingomyelin.

Immune cells are primary targets of PFTs and must balance a complex response to toxin challenge. While immune cells possess mechanisms to repair membrane damage, the disruption and ion imbalance caused by PFTs can trigger a stress response that may ultimately lead to cell death.^29^ To assess this, we examined the cytotoxic effects of Lm supernatant on the monocytic cell line THP-1. The Lm supernatant exhibited cytotoxicity, with increased cell death observed under acidic conditions (Figure 2A and B. A reduction in THP-1 cell death was observed following the addition of Ch:SM-liposomes (Figure 2C and D). When supernatant volumes were adjusted to account for cytotoxic activity, the protective dose of Ch:SM-liposomes remained consistent. Protection plateaued with 10%-20% of the cells remaining dead, and further increases in the Ch:SM-liposome concentration did not improve the protection (Figure S1C). Cholesterol-free (SM-only) liposomes failed to protect the THP-1 cells (Figure S1D), indicating that the neutralizable toxic components in the supernatant have a high affinity for cholesterol, while the remaining toxins likely do not rely on cholesterol or sphingomyelin as receptors.

Residual cell death may result from an immune overreaction to pathogen-associated molecular patterns (PAMPs), which can activate programmed cell death pathways.^30,31^ This overreaction can lead to excessive cytokine production, a phenomenon known as cytokine storm.^32^ We measured cytokine production in THP-1 cells treated with Lm supernatant and observed a dose-dependent increase. This response peaked at a 10-fold increase in IL-8, IL-10 and TNF-*α* expression compared with controls. IL-8 levels were significantly higher in cells treated with supernatant than in those treated with boiled supernatant (1 h at 99 °C, Figure S3A). However, the IL-10 and TNF-*α* levels were similar under both conditions (data not shown). Treatment with Ch:SM-liposomes reduced IL-8 expression to baseline levels (Figure S3B). Notably, the liposomal nanotraps did not induce cytokine expression on their own, confirming prior findings that the composition of naturally occurring lipids is safe and well tolerated (Figure S3B).

### Ch:SM-liposome treatment enhances intracellular bacterial clearance in primary monocyte-derived macrophages

**Figure 3:**
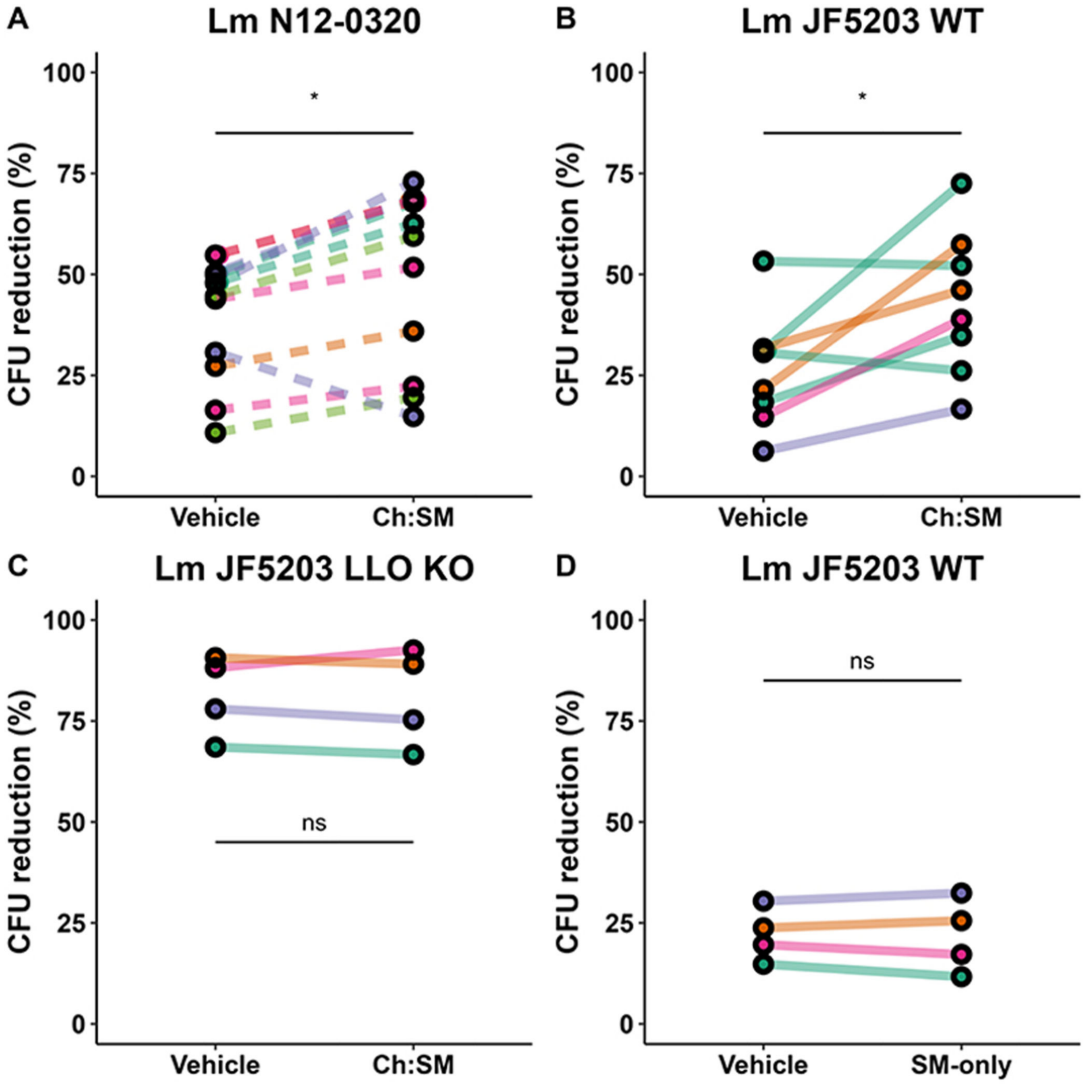
Ch:SM-liposomes enhanced the capacity of macrophages to reduce Lm CFUs. A and B: CFU reduction in macrophages challenged with Lm N12-0320 (A) and Lm JF5203 (B) at an MOI of 10 was enhanced by the addition of 10 µg/mL Ch:SM-liposomes compared with a vehicle control. C: Macrophages reduced CFUs of the Lm LLO knockout strain at a markedly higher rate, with no further enhancement from the addition of Ch:SM-liposomes (C). D: Cholesterol-free liposomes (SM-only) did not affect the capacity of macrophages to reduce Lm CFUs. Each point pair represents an individual experiment. Each color represents a different human monocyte donor. A nonparametric paired Wilcoxon signed-rank test was used as a statistical test; *, *P* < 0.05; ns, not significant. Ch, cholesterol; KO, knockout; LLO, listeriolysin O; Lm, *Listeria monocytogenes*; SM, sphingomyelin.

Since a key stage of the Lm life cycle involves intracellular replication, during which it is able to escape the phagosome before lysosomal fusion, we investigated whether liposomal nanotraps can help immune cells prevent this replication. Monocyte-derived macrophages from human donors treated with low concentrations (10 µg/mL) of cholesterol-containing liposomes and challenged with live Lm, showed a 15%-20% reduction in intracellular Lm CFU counts after 2 hours (Figure 3A and B). Increasing the liposome concentration did not significantly enhance this effect (data not shown). The reduction in CFU was similar for both Lm strains tested, and phagocytosis rates remained unchanged with liposome treatment (data not shown). Rigorous washing between steps ensured that only intracellularly released toxins were involved in this process. An enhanced bacterial clearance effect was also observed in a bovine macrophage cell line (Figure S4). LLO is essential for phagosomal escape by Lm.^33^ When macrophages were challenged with an LLO knockout strain, the CFU reduction efficiency increased, and the addition of Ch:SM-liposomes did not further enhance CFU reduction (Figure 3C). Similarly, the use of SM-only liposomes did not significantly impact the CFU reduction capacity of macrophages (Figure 3D). These results indicate that the enhanced bacterial clearance observed with Ch:SM-liposomes is cholesterol-dependent and likely results from the neutralization of LLO.

### Ch:SM-liposome treatment enhances neutrophil capacity to limit Lm growth

**Figure 4:**
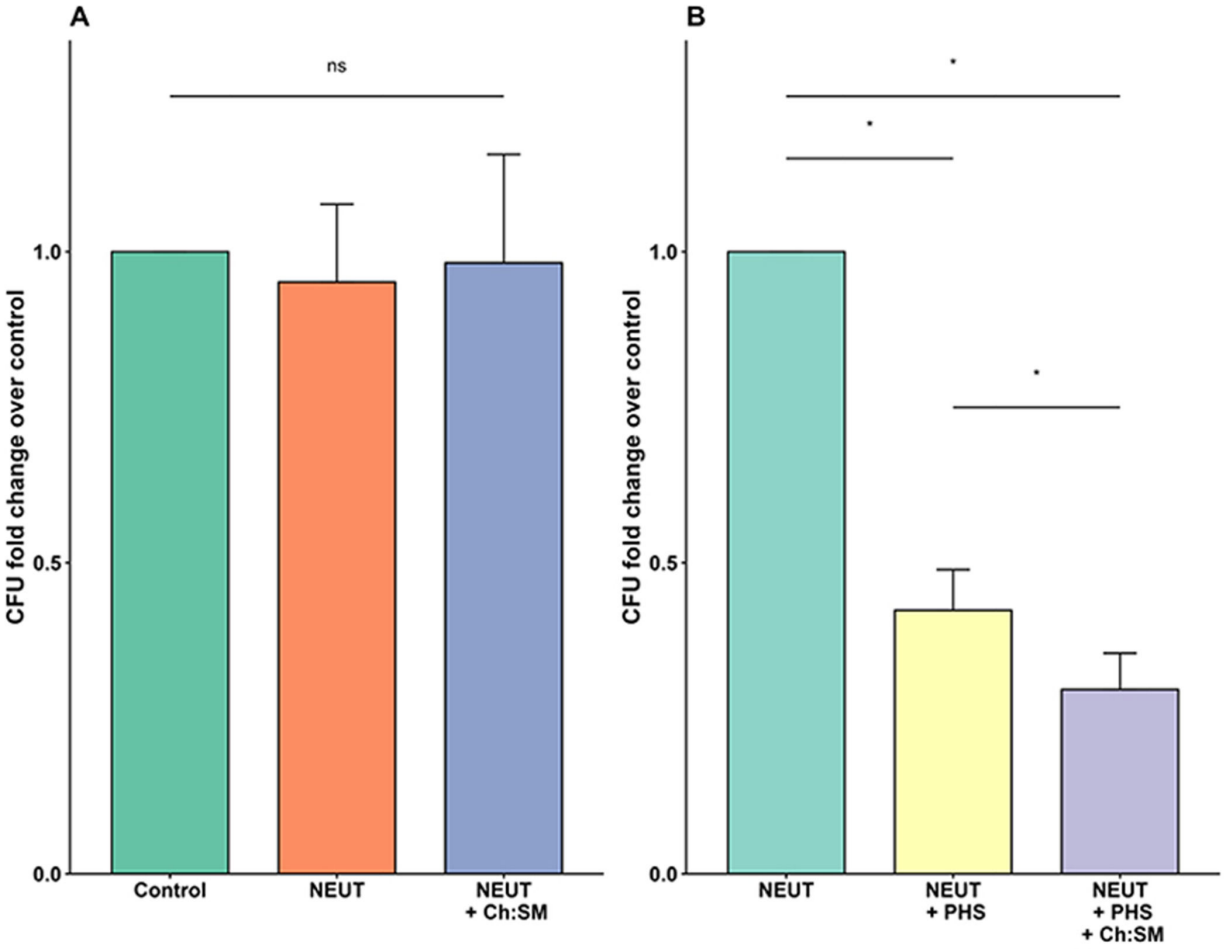
Ch:SM-liposomes enhanced the ability of primary human neutrophils to restrict Lm growth. Primary human neutrophils were incubated with Lm ATCC 19115 bacteria at an MOI of 10 in opsonophagocytic CFU assays. A: In the absence of PHS, neutrophils alone were unable to inhibit Lm ATCC 19115 growth, even with the addition of Ch:SM-liposomes, compared with a bacteria only control. B: The addition of PHS (10%, 30 min, 37°C) significantly reduced Lm ATCC 19115 growth and this reduction was further enhanced by the addition of 50 µg Ch:SM-liposomes. N=4, a different blood donor was used for each replicate. The statistical analysis was performed in R (r-project.org/) using a one-way ANOVA followed by a post hoc Tukey HSD test; *, *P* <0.05; ns, not significant. Ch, cholesterol; Lm, *Listeria monocytogenes*; NEUT, primary human neutrophil; PHS, pooled human serum; SM, sphingomyelin.

In addition to macrophages, neutrophils play a vital role in controlling bacterial infections by employing mechanisms such as reactive oxygen species production (respiratory burst), degranulation of antimicrobial proteins, and formation of neutrophil extracellular traps (NETs) to capture and neutralize pathogens.^34,35^ Like macrophages, neutrophils can also phagocytose and kill bacteria intracellularly. We evaluated the impact of Ch:SM-liposomes on neutrophil-mediated antibacterial activity. Under serum-free conditions, neutrophils were unable to significantly inhibit the growth of Lm ATCC 19115, as the CFU counts after 60 minutes were comparable to the bacteria-only control. The introduction of Ch:SM-liposomes did not result in any significant change in bacterial growth under these serum-free conditions (Figure 4A). However, when Lm were preincubated with 10% pooled human serum for 30 minutes at 37 °C, a marked increase in neutrophil-mediated inhibition of Lm growth was observed (Figure 4B). This inhibitory effect was further amplified by the addition of 50 µg of Ch:SM-liposomes (Figure 4B), mirroring the results observed in macrophages (Figure 3). CFU counts before neutrophil addition confirmed that bacterial numbers were consistent across all groups, indicating that neither the liposomes nor the serum exerted direct bactericidal effects in isolation (data not shown). Gentamicin was not added for this experiment to study the combined impact of Ch:SM-liposomes on both extra- and intracellular Lm. These results imply that Ch:SM-liposomes were able to neutralize extracellular LLO, providing additional protection of neutrophils.

### Bacteria and liposomal nanotraps colocalized after phagocytosis by primary human macrophages

**Figure 5:**
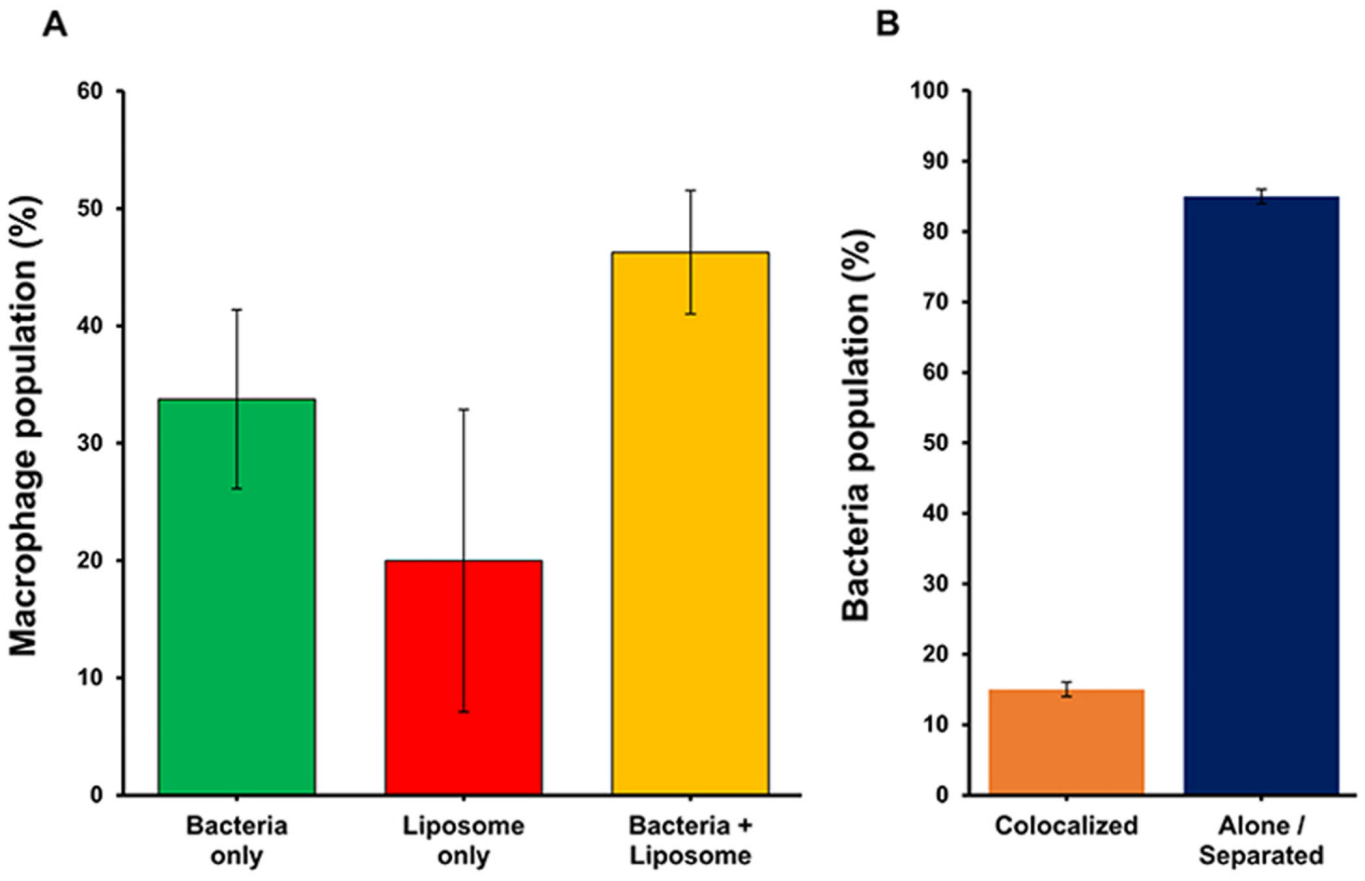
Confocal microscopy images showing colocalization of liposomes and bacteria within macrophages. Monocyte-derived macrophages were incubated with fluorescently labeled liposomes (Ch:SM:DiD, 500 µg) and challenged with Lm JF5203-GFP (MOI = 10). A: Three macrophage populations of similar proportions were identified based on phagocytic content: Phagocytosis of Lm bacteria only, liposome only or both bacteria and liposomes. B: In macrophages that phagocytosed both liposomes and bacteria, most bacteria did not colocalize with Ch:SM-liposomes. Counts were made on > 100 cells. Ch, cholesterol; DiD, DiC18, 1,1'-dioctadecyl-3,3,3',3'-tetramethylindodicarbocyanine, 4-chlorobenzenesulfonate salt; Lm, *Listeria monocytogenes*; SM, sphingomyelin.

We hypothesize that the reduction in intracellular CFUs observed with Ch:Sm-lipopsome treatment could be due to cophagocytosis of liposomes and bacteria in the same phagosome. This colocalization may allow the liposomes to neutralize LLO within the vacuole, preserving vacuole integrity and enabling fusion with lysosomes, ultimately leading to more effective CFU reduction. To test this hypothesis, we labeled Ch:SM-liposomes with the fluorescent dye DiD (DiC18, 1,1'-dioctadecyl-3,3,3',3'-tetramethylindodicarbocyanine, 4-chlorobenzenesulfonate salt) and then coincubated primary human macrophages with Ch:SM:DiD-liposomes and green fluorescent protein (GFP)-expressing Lm. Confocal microscopy (Figure S5A) revealed that similar proportions of macrophages phagocytosed only bacteria (Figure S5B), only liposomes (Figure S5C) or both (Figure S5D), supporting the possibility that colocalization of liposomes and bacteria may contribute to enhanced bacterial clearance. Although bacteria and liposomes are often observed within the same cell (Figure 5A), only about 15% of bacteria colocalize directly with liposomes. The majority of bacteria are separated from liposomes by distances larger than the typical phagosome diameter (Figure 5B). Of note, the 15% colocalization rate aligns with the degree of CFU reduction efficiency observed in the macrophage assays. These findings suggest that the improved macrophage CFU reduction efficiency conferred by liposomal nanotraps is unlikely to be solely due to toxin neutralization during instances where both bacteria and liposomes are phagocytosed into the same vacuole. However, it is worth noting that the colocalization frequency may be underestimated, since the signal emitted by individual liposomes could fall below the detection threshold, meaning that only liposomal clumps are detected.

## Discussion

Bacterial infections account for millions of deaths each year,^36^ and increasing emergence of antibiotic resistance limits the effectiveness of available treatments.^37^ Lm infection can cause severe complications, including meningitis or miscarriage. Here we demonstrate that cholesterol-containing liposomes can neutralize the cytotoxic potential of Lm clinical isolates. By employing an antivirulence approach that targets pathogen virulence factors rather than directly killing bacteria, our goal is to protect host cells and fight infections while reducing the likelihood of developing resistance.

LLO, the major virulence factor of Lm, plays multiple key roles in the bacterial life cycle.^18^ Intracellularly, LLO facilitates phagosomal escape, while extracellularly, it helps breach epithelial barriers and damages immune cells. Host defenses, such as membrane repair and neutrophil metalloproteinase-8 degradation, can counteract LLO, but excessive pore formation leads to cell death.^38^ In this study, we used cholesterol-containing liposomes as active antitoxin agents. These liposomal nanotraps bind toxins with high affinity, outcompeting host membranes for interaction. We showed that these liposomes almost completely neutralized the hemolytic and cytotoxic effects of Lm-secreted toxins at both neutral and acidic pH levels. The requirement for cholesterol in the liposome formulation and neutralization at acidic pH suggests that LLO is a key target. LLO-mediated pore formation is crucial for the internalization of Lm into nonphagocytic cells, and LLO-deficient strains exhibit significantly reduced intracellular loads, highlighting the importance of LLO neutralization.^19^

Despite the strong neutralization of LLO by Ch:SM-liposomes, minor residual cytotoxicity was observed in THP-1 cells, likely due to non–pore-forming toxins. Antigens such as lipoteichoic acid or flagellin, which are recognized by Toll-like receptor (TLR) 2 and TLR5,^39^ may trigger a stress response leading to cell death,^30,31^ even though the pro-inflammatory cytokines IL-8 and TNF-*α* were only slightly upregulated. This suggests that Ch:SM-liposomes are highly effective against pore-forming toxins but may not neutralize all virulence factors.

When Ch:SM-liposomes were added to live Lm bacteria in macrophages, we observed a significant reduction in intracellular cultivable bacteria. This effect was cholesterol-dependent, likely due to LLO neutralization within the phagosome. We found that both bacteria and liposomes can be cophagocytosed, although the frequency of colocalization was low. Given that LLO prefers cholesterol-rich targets, it is plausible that liposomal nanotraps within the phagosome outcompete the host-cell membrane, preserving vacuole integrity and preventing Lm escape. The low detection rate of individual liposomes may lead to an underestimation of their colocalization frequency, yet they effectively neutralized Lm toxins even at pH 5.5, potentially preventing Lm escape before phagosome-lysosome fusion. While the mechanisms underlying Ch:SM-liposome–mediated enhancement of bacterial clearance are not fully understood, further research is needed to elucidate whether additional mechanisms contribute to this antibacterial effect, particularly since higher liposome concentrations did not further improve CFU reduction in this study. In addition, further experiments will be required to assess the potential protective effects of liposomal nanotraps in vivo.

Our study underscores the potential of Ch:SM-liposomes as a novel antivirulence strategy against Lm infections. These liposomal nanotraps not only neutralize Lm toxins, protecting host cells against extracellular damage, but also exhibit intracellular protection, inhibiting Lm at various stages of its life cycle. This dual action can help mitigate severe complications such as meningitis, as PFTs are known to breach the blood-brain barrier.^40,41^ For example, *Streptococcus pneumoniae* secretes pneumolysin, a toxin similar to LLO, which increases blood-brain barrier permeability and facilitates central nervous system invasion.^42^ In addition, Lm can exploit macrophages as “Trojan horses” to cross the blood-brain barrier during infection.^43,44^

## Conclusions

We provide evidence that liposomal nanotraps can neutralize LLO both intracellularly and extracellularly, potentially impeding pathogen spread through both mechanisms. Given their demonstrated safety and broad-spectrum potential, liposomal nanotraps could serve as valuable adjuncts to standard therapies, bolstering the integrity of the host immune response against PFT-expressing bacterial pathogens.

## Materials and methods

### Bacterial culture and strains

The Lm strains used in this study included the following clinical isolates: Lm N12-0320 (serotype 4b, CC4, lineage 1)^45^ and JF5203 (ST1, CC1, lineage 1, RefSeq NZ_LT985474.1).^46,47^ The reference strain Lm ATCC 19115, along with a GFP-expressing variant and an LLO knockout mutant of JF5203,^48,49^ were also included. The bacterial strains are listed in Table S1. Lm was cultured overnight on brain heart infusion (BHI, Sigma-Aldrich) agar plates at 37 °C. A single colony was picked to inoculate BHI broth, where it was grown overnight at 37 °C with shaking at 220 rpm. Cultures were diluted in fresh BHI and incubated until reaching mid-log phase (OD_600_ 0.4-0.6) for live bacteria macrophage challenges or the stationary phase (OD_600_ 0.8-1.2) for supernatant isolation. The supernatants were made bacteria-free by passage through a 0.45 µm filter (Sarstedt), titrated to the desired pH (5.5, 6.5, 7, 7.5), aliquoted, and stored at -80 °C. Unless otherwise specified otherwise, the supernatants were “activated” with 1 mM DTT for 15 minutes at room temperature before use.

### Liposome production

Egg sphingomyelin and cholesterol were purchased from Avanti Polar Lipids and DiD dye from Thermo Fisher. The liposomes were composed of pure sphingomyelin, cholesterol-sphingomyelin (66 mol% cholesterol) or cholesterol-sphingomyelin-DiD (65.9 mol%, 34 mol%, 0.1 mol%, respectively). Mol% denotes the molar percentage—the relative percentage of molecules of each lipid type in the total lipid mixture. Lipids were dissolved in chloroform (10 mg/mL) and DiD in ethanol (1 mg/mL) before mixing. The lipid mixture was dried in a vacuum desiccator (Sigma-Aldrich), then resuspended in phosphate-buffered saline at the desired concentration. Liposomes were formed by sonication (20 min on ice at 5 × 10% cycles, maximal power, Bandelin Sonoplus). Liposome sizes were measured with a NanoSight NS300 (Malvern Panalytical) for Ch:SM-liposomes (108 ± 7 nm) and SM-liposomes (133 ± 6 nm). The liposomes were kept in the dark at 4 °C until further use.

### Hemolysis assay

Bacterial supernatants were serially diluted in 96-well plates and mixed 1:1 with a 2% suspension of human erythrocytes in PBS (final reaction volume = 200 μL), under an approved ethics protocol (P_103 - Interregionale Blutspende SRK AG, Bern, Switzerland). Reactions were conducted at pH 5.5, 6.5 and 7.5 using MES (Applichem) or Tris (Merck) buffers with 0.154 nM NaCl. For protection experiments, liposomal nanotraps were serially diluted in PBS and mixed with erythrocytes. The hemolytic reaction was initiated by adding bacterial supernatant (final reaction volume = 200 μL), followed by incubation at 37 °C for 2 hours. After centrifugation (5 min at 4000 rpm) the erythrocyte pellets were lysed with dH_2_O, and hemoglobin was quantified by measuring the absorbance at 450 nm (ELx808 microplate reader, BioTek). The percentage of hemolysis was normalizing against controls (0% lysis in PBS, 100% lysis in dH_2_O).

### Cell culture, monocyte isolation and macrophage differentiation

Human THP-1 monocytic cells (TIB-202, ATCC) were cultured in RPMI 1640 medium (Gibco, Life Technologies) supplemented with 10% FBS and 1% penicillin-streptomycin (PS) (Gibco, Life Technologies). Peripheral blood mononuclear cells were isolated from human buffy coat using a Lymphoprep gradient (Serumwerk Bernburg AG), under an approved ethics protocol (P_103 - Interregionale Blutspende SRK AG, Bern, Switzerland). Monocytes were frozen in liquid nitrogen and differentiated into macrophages using macrophage colony-stimulation factor (Biolegend) 7 days before the experiments.^50^ Bovine macrophages were cultured in DMEM + PS + FBS at 37 °C with 5% CO_2_.

### Cell survival

THP-1 cells (5 × 10^4^) were added to air permeable tubes (Sarstedt). For cytotoxicity assays, bacterial supernatants were serially diluted and mixed with cells. Liposomal nanotraps were serially diluted for protection assays, and bacterial supernatant was added to initiate the reaction (final volume = 500 µL). After 3 hours of incubation at 37 °C with 5% CO_2_, the cells were washed, resuspended and incubated for 3 to 5 days. Surviving cells were counted with a CellDrop (DeNovix). The results were normalized to those of the PBS controls (0% cell death), with the positive control verifying cytotoxic activity.

### Measurement of the reduction in intracellular bacteria in macrophages

Primary macrophages were seeded on two 24-well plates (1 × 10^5^ cells/well). Bacteria were washed and diluted in RPMI 1640 to reach an MOI of 10 bacteria to 1 macrophage. Either PBS (negative control), Ch:SM-liposomes or SM-liposomes were added to the wells. A centrifugation step of 5 minutes at 110 × *g* increased the contact between bacteria and macrophages and facilitated phagocytosis. The plates were incubated at 37 °C with 5% CO_2_ for 30 minutes. Each well was washed with warm PBS and culture medium was added with 20 µg/mL gentamicin to kill the remaining extracellular bacteria. The plates were incubated at 37 °C with 5% CO_2_. One plate (T0) was taken out after 15 minutes and the other (T1) after 2 hours. The wells were washed, and the intracellular bacteria were released from the macrophages using a 0.05% Triton X-100 solution. The lysate was plated on BHI agar plates and incubated at 37 °C overnight for CFU counting. The correct MOI was verified by plating the inoculum. Macrophages without bacteria were added as controls. T0 corresponded to the number of bacteria that were phagocytosed. T1 corresponded to the number of bacteria alive after lysosome fusion had occurred. The macrophage CFU reduction capacity (%) was calculated as the percentage difference between T0 and T1.

### Primary human neutrophil opsonophagocytic cultivable bacteria count assays

Primary human neutrophils were isolated from healthy human donors with consent under protocols approved by the UC San Diego Institutional Review Board (Protocol #131002) [32] using PolymorphPrep (Cosmo Bio) per the manufacturer’s recommendation. Neutrophils were enumerated on a hemocytometer (Hausser Scientific) after lysis of residual red blood cells and immediately added to the wells. Lm ATCC 19115 were incubated with or without 10% pooled human sera (to opsonize bacteria) and 50 µg of Ch:SM-liposomes for 30 minutes at 37 °C as indicated. Serum-coated bacteria were added to 2 × 10^5^ human neutrophils in triplicate at an MOI of 10. After a brief spin (2 min, 500 × *g*) to increase bacteria-neutrophil contact, the plates were incubated for 1 hour at 37 °C with 5% CO_2_, the neutrophils were lysed in dH_2_O for 3 minutes, and the lysates were serially diluted in PBS, plated on BHI agar and incubated overnight at 37 °C for CFU enumeration.

### Quantitative real-time PCR

THP-1 cells were activated with 5 ng/mL of phorbol 12-myristate 13-acetate (PMA, Sigma-Aldrich) for 24 hours in 1-mL RPMI 1640 + PS + FBS. The cells were challenged with 200 µL of bacterial supernatant with or without liposomes, boiled supernatant, or medium only. After gentle mixing, the plates were incubated for 3 hours at 37 °C with 5% CO_2_. Total RNA was isolated using the RNeasy Micro Kit (Qiagen) and quantified using a NanoDrop (Thermo Fisher). Complementary DNA (cDNA) was synthesized from isolated RNA using the High-Capacity cDNA Reverse Transcription Kit (Applied Biosystems) following the manufacturer’s instructions. Quantitative real-time PCR primers were generated using the Primer 3 design software (listed in Table S2). The SYBR Green detection system was used per the manufacturer’s instructions (Quanta Biosciences). Relative expression was quantified in a 96-well format on a QuantStudio 6 (Thermo Fischer Scientific). Data were analyzed using R software (version 4.4.0), and GAPDH was used as the normalization gene to calculate the level of mRNA expression with the 2-ΔΔCT method.^51^

### Confocal microscopy

Monocyte-derived macrophages were differentiated as mentioned previously and seeded on glass 8-well slides (Sarstedt) at 1 × 10^5^ cells/well. The macrophages were infected with washed Lm bacteria in exponential phase (OD_600_ 0.4-0.6) at an MOI of 10 and incubated with fluorescent Ch:SM:DiD-liposomes (10-1000 µg/mL). After 5-minutes centrifugation at 110 × *g* to maximize contact, the mixture was incubated for 30 minutes without antibiotics, washed, incubated for 15 minutes with gentamicin (20 µg/mL), washed, and fixed with 4% paraformaldehyde at room temperature for 15 minutes. Images were taken using an LSM 880 confocal microscope (Carl Zeiss) with a 63× oil immersion objective and imaged with Zen software SMART using a channel for GFP, one for DeepRep, and widefield. Images were processed on Napari (version 0.4.18)^52^ and counted independently by two researchers.

## Supplementary Material

Supplementary Materials

## Data Availability

The datasets generated during and/or analyzed during the current study are available from the corresponding author on reasonable request.
