# Supplementary material for: Liposomal Nanotraps Neutralize Listeria monocytogenes Toxins to Enhance Macrophage Viability and Antibacterial Capacity
Source: Infect Microbes Dis. Author manuscript; Available in PMC 2025 Jun 9. (PMC7617752; doi:10.1097/IM9.0000000000000177)
Supplement: Supplementary Materials [file EMS205988-supplement-Supplementary_Materials.pdf]

Supplementary Digital Contents

Table S1. List of the bacterial strains used in the study.

Species	Strain	Characteristic	Ref.
Listeria monocytogenes	N12-0320	Clinical isolate, serotype 4b, CC4, lineage 1	45
Listeria monocytogenes	19115	Reference strain	ATCC
Listeria monocytogenes	JF5203	Veterinary isolate (bovine), ST1, CC1, lineage 1, RefSeq NZ_LT985474.1	46,47
Listeria monocytogenes	JF5203-GFP	Constitutively expressing GFP	48
Listeria monocytogenes	JF5203-LLO KO	Knock out of the *hly* gene responsible for LLO synthesis	49

ACCEPTED

Table S2. Primers used for quantitative real-time PCR.

Gene	Accession number	Sequence (5’ → 3’)
IL-8	NM_001354840.3	Fwd: ACTGAGAGTGATTGAGAG
		Rev: AACCCTCTGCACCCAGTT
TNF-α	NM_000594.4	Fwd: AGCCTCTTCTCCTTCCTGATCGTG
		Rev: GGCTGATTAGAGAGAGGTCCCTGG
IL-10	NM_000572.3	Fwd: GCCTAACATGCTTCGAGATC
		Rev: TGATGTCTGGGTCTTGGTTC
GAPDH	NM_001357943.2	Fwd: GAAATCCCATCACCATCTTCCAGG
		Rev: CGCGGCCATCACGCCACAGTTTCC

ACCEPTED

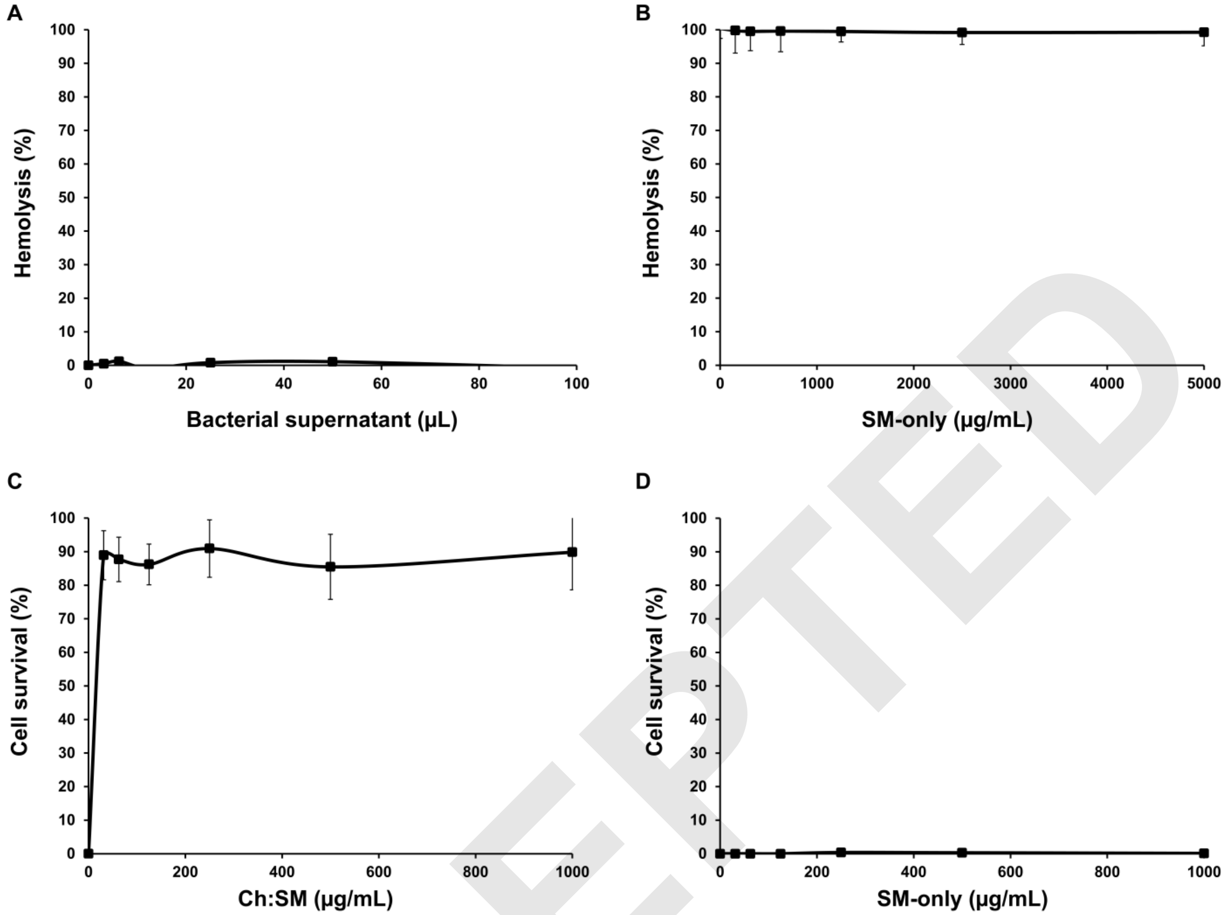

Figure S1. A: Lm JF5203 supernatant shows no hemolytic activity in absence of DTT at pH 7. B: The addition of a cholesterol-free SM-only liposome to an Lm JF5203 supernatant challenge does not protect erythrocytes at pH 6.5. C: High concentrations of Ch:SM-liposomes protect THP-1 cells against Lm toxins with a plateau at approximately 90% cell survival. D: The addition of a cholesterol-free SM-only liposome to an Lm JF5203 supernatant challenge does not protect THP-1 cells at pH 6.5. Error bars = mean ± SD. n ≥ 3. Ch, cholesterol; DiD, DiC18, 1,1'-dioctadecyl-3,3,3',3'-tetramethylindodicarbocyanine, 4-chlorobenzenesulfonate salt; Lm, *Listeria monocytogenes*; SM, sphingomyelin.

ACCEPTED

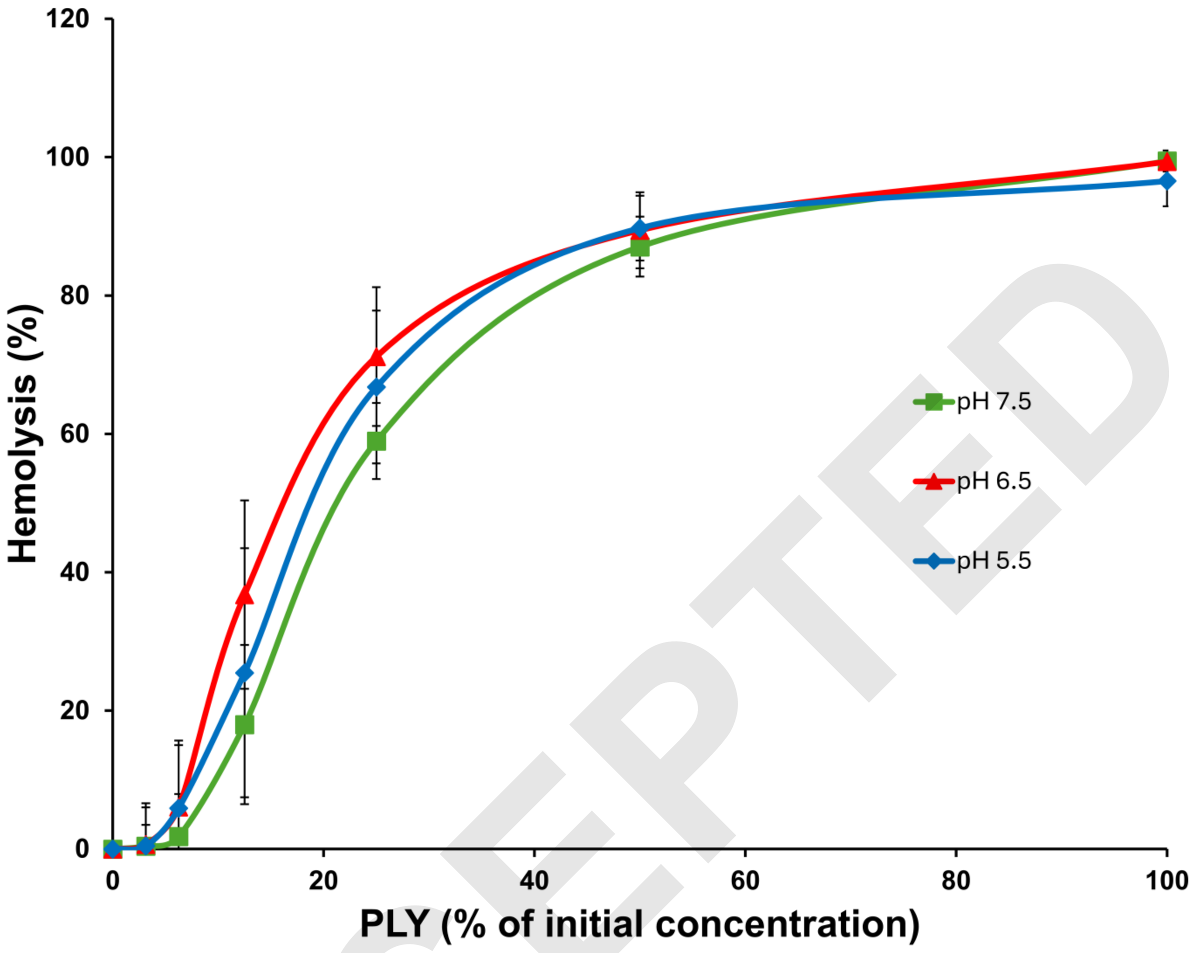

ACCEPTED

Figure S2. The hemolytic activity of the CDC PFT pneumolysin was not increased at acidic pH. Error bars = Mean ± SD. n ≥ 3. CDC, cholesterol-dependent cytolysin; PFT, pore-forming toxins; PLY, pneumolysin.

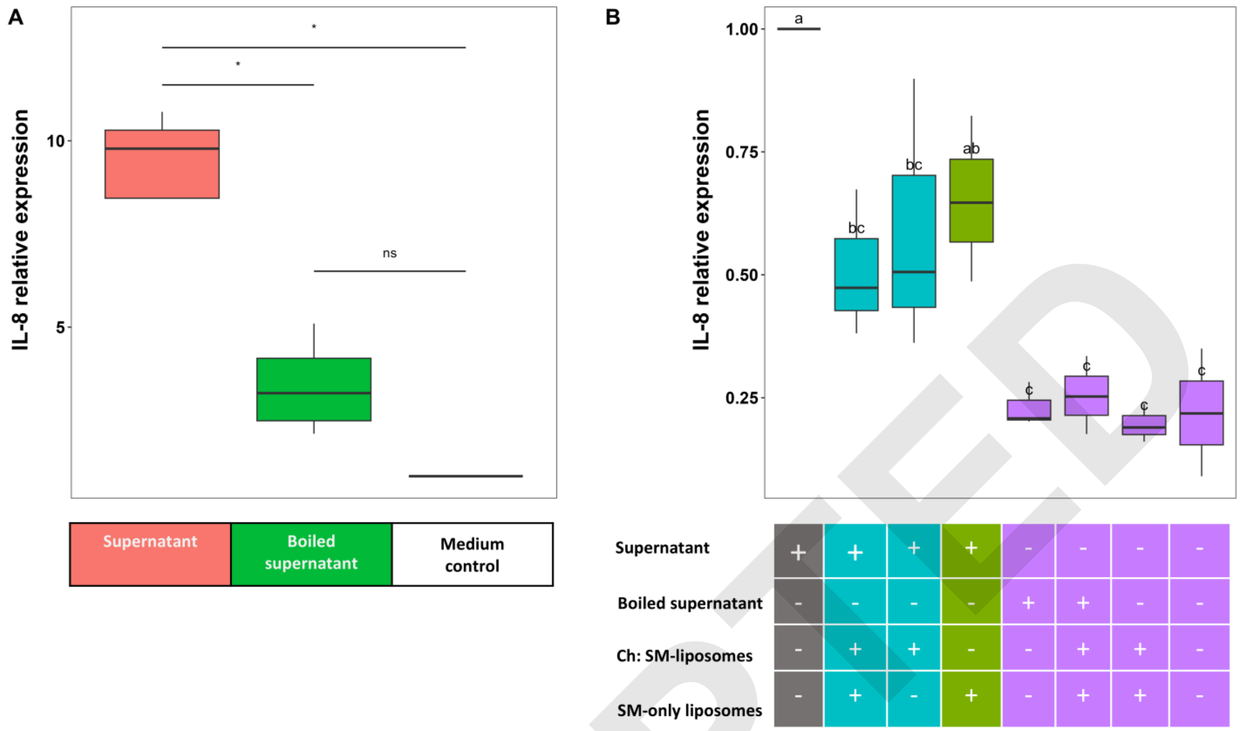

Figure S3. A: Lm N12-0320 supernatant upregulated IL-8 mRNA expression in activated THP-1 cells. B: The addition of Ch:SM-liposomes lowered the stimulation to negative control levels. SM-only liposomes did not differ significantly from the level of supernatant alone. Neither Ch:Sm- or SM-only-liposomes were pro-inflammatory. Normalization was done on BHI medium control (A) or untreated supernatant (B). Conditions that do not share a letter show statistically significant difference (ex: The difference between "bc" and "c" is not statistically significant but the difference with "a" is). N $\geq$ 3. BHI, brain heart infusion; Ch, cholesterol; Lm, *Listeria monocytogenes*; SM, sphingomyelin.

ACCEPTED

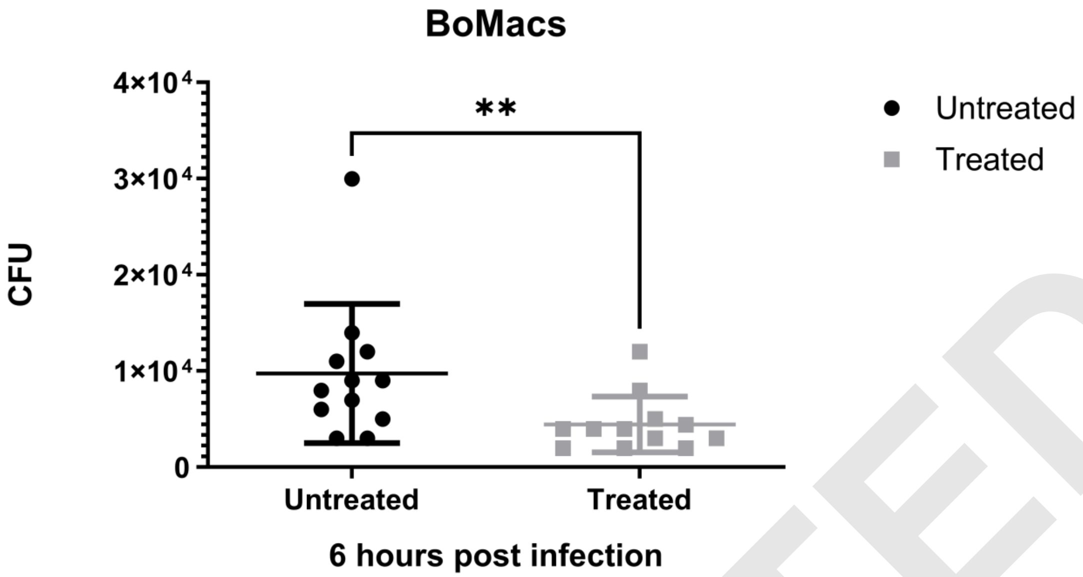

Figure S4. **Ch:SM-liposomes enhance the Lm infection control of bovine macrophages.** The complete gentamicin protection protocol was described previously.[53] Briefly, bovine macrophages (BoMacs) were challenged with Lm JF5203 at an MOI of 10. When treated with 1 mg/mL of Ch:SM-liposomes, BoMacs showed reduced CFUs compared with untreated BoMacs. The results represent four experiments with three technical replicates each. A non-parametric unpaired Mann-Whitney U test was used as a statistical test; **, P <0.01. Ch, cholesterol; Lm, *Listeria monocytogenes*; SM, sphingomyelin.

ACCEPTED

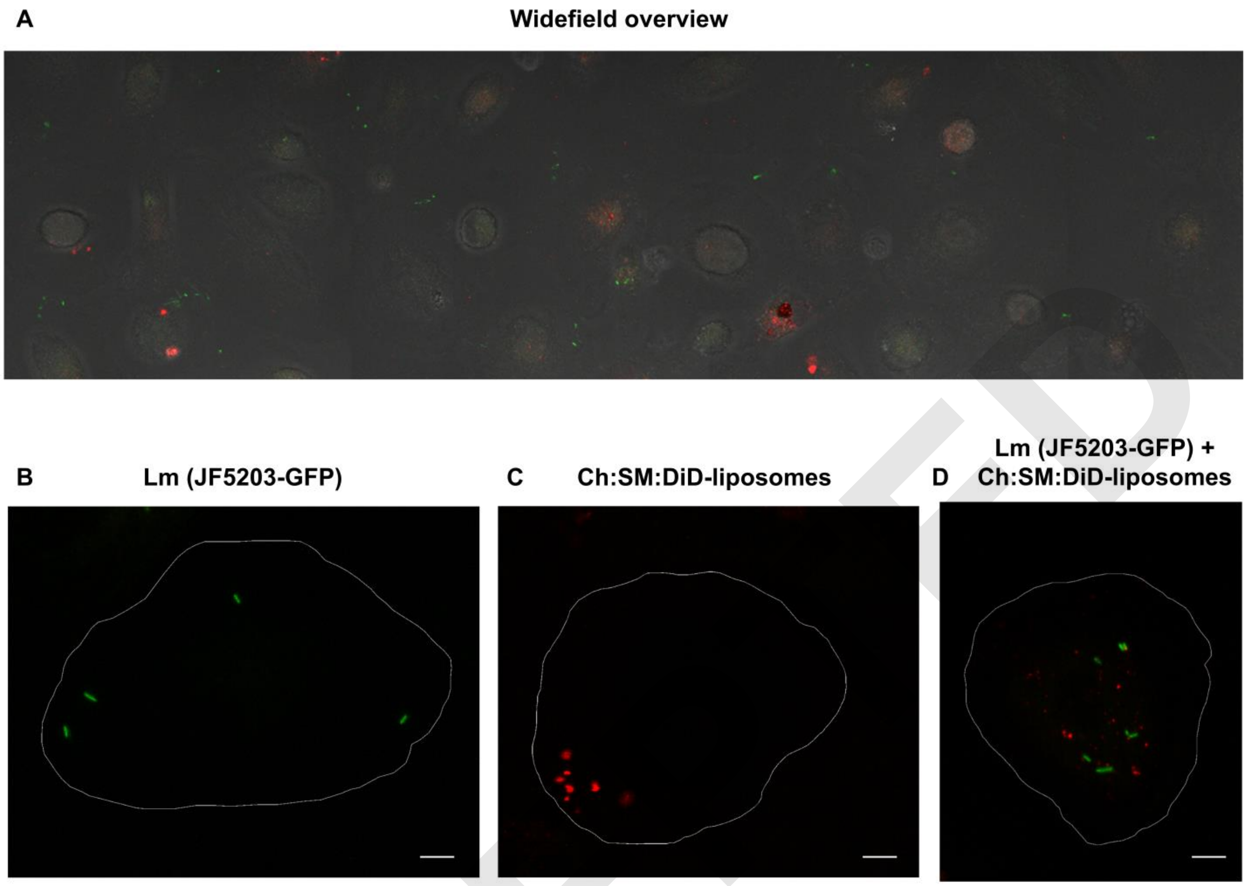

Figure S5. **Monocyte-derived macrophages were incubated with fluorescently labeled liposomes (Ch:SM:DiD, 500 µg) and challenged with Lm JF5203-GFP (MOI = 10).** A: Confocal microscopy images were acquired via systematic and unbiased tile imaging. B, C and D: Three macrophage populations of similar proportions were identified based on phagocytic content: Phagocytosis of Lm bacteria only (B), liposome only (C) or both bacteria and liposomes (D). Cell borders were determined based on widefield images. Scale bars = 2 µm. Ch, cholesterol; DiD, DiC18, 1,1'-dioctadecyl-3,3,3',3'-tetramethylindodicarbocyanine, 4-chlorobenzenesulfonate salt; Lm, *Listeria monocytogenes*; SM, sphingomyelin.

ACCEPTED
